# *N*-Acetylcysteine Nanocarriers Protect against Oxidative Stress in a Cellular Model of Parkinson’s Disease

**DOI:** 10.3390/antiox9070600

**Published:** 2020-07-09

**Authors:** Leah Mursaleen, Brendon Noble, Stefanie Ho Yi Chan, Satyanarayana Somavarapu, Mohammed Gulrez Zariwala

**Affiliations:** 1 School of Life Sciences, University of Westminster, 115 New Cavendish Street, London W1W 6UW, UK; w1655446@my.westminster.ac.uk (L.M.); b.noble@westminster.ac.uk (B.N.); 2 Department of Pharmaceutics, UCL School of Pharmacy, 29-39 Brunswick Square, London WC1N 1AX, UK; ho.chan.16@ucl.ac.uk (S.H.Y.C.); s.somavarapu@ucl.ac.uk (S.S.); 3 The Cure Parkinson’s Trust, 120 New Cavendish St, Fitzrovia, London W1W 6XX, UK

**Keywords:** *N*-acetylcysteine, oxidative stress, Parkinson’s disease, neurodegeneration, antioxidant capacity, deferoxamine, iron

## Abstract

Oxidative stress is a key mediator in the development and progression of Parkinson’s disease (PD). The antioxidant *N*-acetylcysteine (NAC) has generated interest as a disease-modifying therapy for PD but is limited due to poor bioavailability, a short half-life, and limited access to the brain. The aim of this study was to formulate and utilise mitochondria-targeted nanocarriers for delivery of NAC alone and in combination with the iron chelator deferoxamine (DFO), and assess their ability to protect against oxidative stress in a cellular rotenone PD model. Pluronic F68 (P68) and dequalinium (DQA) nanocarriers were prepared by a modified thin-film hydration method. An MTT assay assessed cell viability and iron status was measured using a ferrozine assay and ferritin immunoassay. For oxidative stress, a modified cellular antioxidant activity assay and the thiobarbituric acid-reactive substances assay and mitochondrial hydroxyl assay were utilised. Overall, this study demonstrates, for the first time, successful formulation of NAC and NAC + DFO into P68 + DQA nanocarriers for neuronal delivery. The results indicate that NAC and NAC + DFO nanocarriers have the potential characteristics to access the brain and that 1000 μM P68 + DQA NAC exhibited the strongest ability to protect against reduced cell viability (*p* = 0.0001), increased iron (*p* = 0.0033) and oxidative stress (*p* ≤ 0.0003). These NAC nanocarriers therefore demonstrate significant potential to be transitioned for further preclinical testing for PD.

## 1. Introduction

Oxidative stress—damage to proteins, lipids and DNA as a result of toxic reactive oxygen species (ROS)—is a common feature of many neurodegenerative diseases including Parkinson’s disease (PD), where it has been linked to both genetic and sporadic forms of the condition [1,2,3]. Numerous neurotoxins used to model PD, such as 1-methyl-4-phenyl-1, 2, 3, 6-tetrahydropyridine (MPTP) and rotenone, inhibit mitochondrial respiration, resulting in an accumulation of ROS, inducing a form of parkinsonism in both animals and humans [4,5,6,7,8,9,10].

Although ROS are a natural bi-product of oxidative phosphorylation, in PD, ROS production is heightened due to the impairment of antioxidant defence mechanisms (e.g., superoxide dismutase, glutathione peroxidase and catalase) via a reduction in Nrf2-Keap1 signalling responsible for regulating the expression of many endogenous antioxidants [11,12,13,14,15,16,17]. For example, it has been reported that glutathione levels in the substantia nigra of people with PD are reduced by up to 40% compared to healthy controls [18]. This, coupled with excessive levels of free iron present in PD, induces sustained formation of toxic hydroxyl radicals and the resultant oxidative stress drives neuronal dysfunction and ultimately degeneration of dopaminergic neurons within the substantia nigra [11,12,16,19,20,21].

N-acetylcysteine (NAC) is an antioxidant that is approved by the U.S. Food and Drug Administration (FDA) to treat paracetamol overdose and is also marketed as an antioxidant dietary supplement in the US [22]. As a precursor of endogenous glutathione, NAC has long been considered to have potential as a disease-modifying therapeutic for PD. Preclinical studies of NAC in PD models have shown that it is protective against dopaminergic cell death induced by rotenone [23] and MPTP [24,25,26,27]. Clark et al. [28] demonstrated that NAC can also protect against toxic alpha synuclein aggregates in transgenic mice, increasing glutathione levels within 5–7 weeks of treatment. There have also been numerous clinical assessments of NAC for neurological conditions, including PD [13,29,30,31,32]. Although it has been shown that NAC can reach the cerebral spinal fluid following oral administration [31,33], high doses are required, since oral NAC is only 5% bioavailable at therapeutic doses and is largely undetectable following ingestion [13,34]. However, administering high doses of NAC may be undesirable due to the increased likelihood of side effects such as headaches, diarrhoea and vomiting [35,36,37]. Overproduction of glutathione associated with high concentrations of NAC has also been reported to be cytotoxic in dopaminergic neurons [38]. Consequently, it would be of benefit to utilise a delivery system to improve the bioavailability of NAC and enhance access to the brain, thus mitigating the need for administering treatments with high concentrations.

Nanocarriers can enhance the potency, stability, bioavailability and passage across biological membranes of entrapped or associated compounds [39,40,41,42]. Different nanoformulations have been investigated to overcome the low bioavailability and short half-life of NAC but these have mainly been focused upon pulmonary delivery to treat conditions such as acute lung injury and lung cancer [43,44,45]. To our knowledge, no NAC formulations have been investigated in models of neurodegenerative diseases such as PD or developed specifically to target mitochondria which are the principle site for ROS generation and responsible for 90% of total cellular ROS production [12,19,21]. We previously developed a successful nanocarrier delivery system composed of FDA-approved components, the block co-polymer Pluroninc F68 (P68) and the mitochondrial targeting molecule dequalinium (DQA). Our delivery system exhibited many of the advantageous characteristics of polymeric micelles that make them suitable for brain penetrance, for example small particle size (<200 nm) and relatively neutral charge (<10 mV) [42]. Our previous results suggest that combined delivery of antioxidants and iron chelators may provide the most promising strategy to limit the degenerative process in PD due to the dual approach of free radical scavenging and iron chelation to limit detrimental free iron availability [42]. The aim of this study was therefore to utilise the P68 + DQA nanocarriers for NAC alone and in combination with the iron chelator deferoxamine (DFO), and assess their physical characteristics and ability to protect against oxidative stress in a cellular rotenone model of PD.

## 2. Materials and Methods

### 2.1. Materials

Unless otherwise stated, all chemicals were of analytical grade and cell culture grade where applicable. SH-SY5Y cells were purchased from the American Type Culture Collection (ATCC CRL-2266, USA). Methanol (HPLC grade), L-glutamine, foetal bovine serum (FBS), Dulbecco’s Modified Eagle Medium (DMEM) Glutamax®, minimum essential media (MEM), 100 × antibiotic-antimycotic, poloxomer 68 (pluronic F68) and the Pierce BCA protein assay kit were purchased from Fisher Scientific, UK. Dequalinium chloride hydrate (DQA; 95%), Dulbecco’s phosphate-buffered saline (DPBS), *N*-Acetyl-L-cysteine (99%), deferoxamine mesylate salt (92.5%), rotenone (≥95%), protease inhibitor cocktail (PIC, cat no. P8340), thiazolyl blue tetrazolium blue (MTT), dimethyl sulfoxide (DMSO), 2′,7′-dichlorofluorescin diacetate (DCFH-DA), 2,2′-azobis(2-methylpropionamidine) dihydrochloride (ABAP) and 2, 4, 6-tripyridyl-s-triazine and iron(III) chloride hexahydrate were purchased from Sigma-Aldrich, UK. The ferrous sulphate (20% iron), ferrozine, ammonium acetate, ascorbic acid, potassium permanganate and hydrochloric acid used for the ferrozine assay were also purchased from Sigma-Aldrich, UK. The mitochondrial hydroxyl radical detection assay kit (cat no. ab219931) was purchased from Abcam, UK. The ferritin ELISA kit (product code S-22) was from ATI Atlas (Chichester, UK). The TBARS parameter assay kit (product code KGE013) was purchased from R&D Systems, Parameter TM, UK. Experimental reagents were prepared using Milli-Q water (water purified through a 0.22 μm membrane filter with a resistivity of 18.2 MΩ). Sterile filters were from Millex-MP, Millipore, Ireland. Flasks were from Nunc (Denmark) and all culture plates, pipettes, stripettes and eppendorf tubes were from Corning, UK.

### 2.2. Preparation of Micellar Nanocarriers

All nanoformulations were prepared using a modified thin-film hydration method [40,46]. Briefly, P68 and DQA were dissolved in 10 mL of methanol along with NAC alone or in combination with DFO at certain ratios (Table 1) and sonicated for up to 1 min using a VWR ultrasonic cleaner bath USC300T (VWR International Limited, UK). Using a rotary evaporator (Hei-VAP Advantage Rotary Evaporator, Heidolph, Germany) the methanol was then evaporated at 200 rpm and 80 °C, under vacuum until a thin film was obtained. The resultant thin film was hydrated with 10 mL of warmed distilled water and mixed thoroughly at 80 °C for 1–2 min and then sonicated for a further 1 min until the film was fully removed and dispersed in the water. In order to remove any unassociated NAC and DFO, the solution was filtered through a sterile 0.22 μm filter. A proportion of the samples were lyophilized using a Virtis AdVantage 2.0 BenchTop freezedryer (SP Industries, UK) for further analysis. P68 + DQA DFO nanoformulations previously characterised by Mursaleen et al. [42] were prepared as described above and used as a control treatment in the cellular assays.

### 2.3. Size and Surface Charge of the Nanocarriers

The particle size and surface charge of nanocarriers were measured in triplicate using the Zetasizer Nano ZS (Malvern Instruments, UK). Size distribution was measured via photon correlation spectroscopy as Z-Ave hydrodynamic diameter and polydispersity index (PDI). Each formulation was prepared at least six times and measurements were taken in triplicate for each sample.

### 2.4. Determination of Drug Association and Association Efficiency

UV–visible (UV–vis) spectroscopy was employed to study drug association and association efficiency of the nanoformulations based on the calibration curves of the free drugs, as previously described by Mursaleen et al. [42]. Briefly, methanol and water (1:1) were used to dissolve the carrier to release the drug and achieve a theoretical concentration of each drug (1 mg/mL NAC and 20 μg/mL DFO). NAC and DFO content were calculated using UV–vis spectroscopy at 204 and 234 nm, respectively. The percentage of drug association and association efficiency were calculated using the following equations:

Drug association (%) = (determined mass of drug within nanocarriers/mass of drug-associated nanocarriers) × 100(1)

Association efficiency (%) = (determined mass of drug within nanocarriers/theoretical mass of drug within nanocarriers) × 100(2)

### 2.5. Thermal Properties of the Nanocarriers

X-ray diffraction (XRD) patterns were obtained using an X-ray diffractometer for pure NAC, DFO, DQA, P68, lyophilized P68 + DQA formulations of NAC and NAC + DFO to determine the atomic and molecular structure (Rigaku MiniFlex600, Miniflex, Japan). All samples were analysed at room temperature in the angle range 5−35°, with a step size of 0.01° and a scanning rate of 2°/min.

### 2.6. Antioxidant Capacity of the Nanoformulations

The potential antioxidant capacity of drug-associated nanoparticles was determined using the modified ferric ion-reducing antioxidant power (FRAP) assay and compared to the FRAP results of the corresponding free drug, as previously described [40]. To prepare the FRAP reagent, acetate buffer (pH 3.6), tripyridyl triazine, and iron (III) chloride were mixed together. The concentrations of drug associated with the nanoparticles was spectrophotometrically measured using a microtiter plate reader (VersaMax, Molecular Devices, USA) as described above. In order to analyse the antioxidant activity, samples of the formulations and drug stock solutions were added to the FRAP reagent and incubated at 25 °C for 30 min. The absorbance of samples was read at 593 nm. The blank comparator was FRAP reagent without the addition of any treatment. The absorbance values were normalised as Trolox equivalent antioxidant capacity, as Trolox is a recommended baseline antioxidant reference point for multiple antioxidant assays [47,48].

### 2.7. SH-SY5Y Cell Culture

The human neuroblastoma SH-SY5Y cell line was used to create an in vitro model of PD (as previously described by Mursaleen et al. [42] and reviewed in Xicoy et al. [49]). SH-SY5Y cells were grown in DMEM Glutamax®, pH 7.4, supplemented with 10% FBS and 1% antibiotic/antimycotic in a 5% CO_2_ environment at 37 °C. SH-SY5Y cells were thawed and grown in plastic T75 flasks until they reached 70% confluence. Adherent cells were then detached from the surface of the flasks via trypsinisation and seeded at 1,000,000 cells/cm^2^ into well plates at specific numbers according to the bioassay being performed (6-well or 96-well plates).

Based on methods previously described [42,50,51], cells were pre-treated for 3 h with various concentrations of the free, P68 + DQA treatments or corresponding blank formulations before being treated with 100 μM rotenone (in MEM) for 24 h at 37 °C. Rotenone-only and MEM-only treatments, without any pre-treatments, were used as controls.

Where necessary, cells were grown in 6-well plates until confluent and lysed at 4 °C, as previously described by Zariwala et al. [52], using 350 µL ice-cold lysis buffer (50 mM NaOH supplemented with 1 ug/mL protease inhibitor cocktail) whilst rocking gently for 40 min in ice trays on a see-saw rocker at 8 rpm (model SSL3/1, Stuart, UK). Cell lysates were collected using sterile cell scrapers, passed through a 25G needle and aliquoted into microcentrifuge tubes ready for further analysis. For each experiment requiring the use of cell lysate, the total protein content was determined using the Pierce BCA kit following the manufacturer’s protocol (as previously described by Kim et al. [50]), using the bovine serum albumin (BSA) stock (2 mg/mL) provided in the kit as the standard.

### 2.8. Cell Viability Analysis

The protective properties of drug-associated nanocarriers against rotenone-induced reduction in cell viability was assessed using the MTT Assay, as previously described [42]. Briefly, following the 24 h rotenone treatment in 96-well plates, SH-SY5Y cells were incubated with an additional 20 μL of 5 mg/mL MTT DPBS solution for 4 h at 37 °C. Following aspiration, 100 μL of DMSO was added to each well to dissolve the formazan crystals. To ensure that DMSO was mixed well, plates were placed on a MaxQ 4000 benchtop orbital shaker (Thermo Fisher Scientific, UK) at 75 rpm for 15 min and the absorbance was read at 570 nm using a spectrophotometer.

### 2.9. Iron Content Analysis

Cell harvesting and iron absorption and content experiments were carried out using the method previously described by Zariwala et al. [52]. SH-SY5Y cells were grown in 6-well plates and pre-treated with free drug or drug-associated nanoformulations before treatment with rotenone and then lysed as described above.

Total iron was quantified using a modified version of the ferrozine colorimetric assay used in Zariwala et al. [52]. Briefly, 200 μL of 0.1 M HCL was added to 200 μL of sample lysate. The iron standards were prepared using analytical grade FeSO_4_. Samples were then incubated in the dark with 200 μL of iron-releasing agent (1.4 M HCL and 4.5% KMnO_4_ in water) for 2 h at 60 °C under a fume hood. Samples were then cooled to room temperature before being incubated for 30 min with 60 μL of iron detection reagent (6.5 mM ferrozine, 2.5 M ammonium acetate, 1 M ascorbic acid). Equal volumes (200 μL) of the test and standard samples were aliquoted into a 96-well microplate in duplicate and absorbance was read at 550 nm using a microplate reader (VersaMax, Molecular devices, USA).

Cell lysate samples were also assessed for the ferritin concentration using a spectrophotometric ELISA kit. In total, 30 μL of each sample and standard were added into a 96-well plate in duplicate. Incubation steps were carried out as described in the manufacturer’s protocol. Briefly, samples and standards were incubated in 200 μL of the conjugate for 2 h at 195 rpm, 37 °C using a MaxQ 4000 benchtop orbital shaker (Thermo Fisher Scientific, UK). The samples and standards were then washed three times with Milli-Q water before they were incubated with 200 μl of the substrate solution for 30 min at room temperature. Following incubation, 100 μL of potassium ferricyanide solution was added to each sample and standard, and the absorbance was read at 495 and 630 nm using a microplate reader (VersaMax, Molecular devices, USA). All samples were assayed in duplicate. The ferritin and ferrozine concentrations were standardised against the total protein concentration.

### 2.10. Cellular Antioxidant Activity

The cellular antioxidant activity was measured based on the slightly modified method of the original assay developed by Wolfe et al. [53], as previously described [42,54,55]. SH-SY5Y cells were seeded in black-walled, clear-bottom 96-well microplates. Once confluent, cells were washed with DPBS and treated with different concentrations of 200 μL of drug-associated nanocarriers or drug solution for 1 h at 37 °C. Cells were then washed with MEM and treated with 200 μL of the fluorescent probe DCFH-DA (100 μM) and incubated for a further 30 min at 37 °C. Following aspiration, each well was treated 100 μL of prooxidant (600 μM ABAP or 100 μM rotenone) dissolved in MEM. The assay was modified to also test rotenone as the prooxidant in order more closely mimic the oxidative stress present in PD. The fluorescence of the cells in the 96-well plate was read every 5 min for 1 h at 528 and 485 nm emission and excitation (respectively) on the Fluostar Optima Fluorescence Plate Reader. The CAA unit was calculated using the following equation:

CAA unit = 100−(area under the curve (AUC) of the treatment/AUC of control) × 100(3)

AUC = (1 + (RFU1/RFU0) + (RFU2/RFU0))(4)

where RFU0 is the relative fluorescence value of point zero and RFUx is the relative fluorescence of each time point (e.g., RFU5 is relative fluorescence value at minute 5).

### 2.11. Lipid Peroxidation Analysis

The thiobarbituric acid-reactive substances (TBARS) assay was used to assess oxidative stress, specifically lipid peroxidation, by reacting the secondary end product of the oxidation of polyunsaturated fatty acids (malondialdehyde) with thiobarbituric acid (TBA) in a colorimetric reaction to form TBARS [56,57]. Briefly, SH-SY5Y cells were grown in 6-well plates until confluent. Following pre-treatment with the relevant free or nanoformulated conditions and 24 h rotenone treatment, cells were washed once with DPBS and lysed at 4 °C as described above. The TBARS assay was carried out in accordance with manufacturer guidelines (R&D Systems, Parameter TM) and as previously described [42]. Freshly prepared TBA was added to TBARS acid-treated cell lysate which was incubated at 60 °C for 2.5 h. The absorbance of samples was read at 532 nm before and after incubation to estimate the formation of TBARS.

### 2.12. Mitochondrial Hydroxyl Analysis

The mitochondrial hydroxyl radical detection assay is a fluorometric assay which detects intracellular hydroxyl radical using an OH580 probe that selectively reacts with hydroxyl radical present in live cells. Such a reaction generates a red fluorescence signal that can be read at 540/590 nm excitation/emission. The assay was carried out according to the manufacturer’s protocol (ab219931; Abcam, UK). Briefly, SH-SY5Y cells were seeded in black-walled, clear-bottom 96-well microplates until confluent. Cells were then washed with DPBS and treated with different concentrations of drug-associated nanocarriers or free drug at a volume of 200 μL for 3 h at 37 °C. Following this, cells were washed with DPBS and treated with 100 µL of 6.25X OH580 probe for 1 h at 37 °C. A volume of 100 µL of 200 µM rotenone (final concentration 100 µM) was then added to each well and cells were incubated for 24 h at 37 °C. Cells were then washed with DPBS and the fluorescence was read on the Fluostar Optima Fluorescence Plate Reader.

### 2.13. Statistical Analysis

For all experiments, the mean of six replicates was calculated for each treatment, with the data expressed as the mean ± standard deviation (S.D.). The MTT, ferrozine, TBARS, mitochondrial hydroxyl assays and ferritin ELISA results were statistically analysed using one-way analysis of variance (ANOVA) followed by Dunnett’s T3 post hoc test. A two-way ANOVA followed by Tukey’s multiple comparisons post hoc test was used to analyse the FRAP and CAA assays (PRISM software package, Version 8, Graphpad Software Inc., San Diego, USA).

## 3. Results

All drug-associated nanoformulations exhibited high mean association efficiency (93–98%) (Table 1). The drug-associated nanocarriers exhibited a significantly higher mean particle size compared to the blank formulations without actives (*p* < 0.0001) (Table 1). The addition of DFO into the formulation appeared to increase the mean association efficiency of NAC by 5%. The mean size of both the NAC (126 nm) and NAC + DFO (130 nm)-associated P68 + DQA nanocarriers was less than 200 nm (Table 1). The addition of DFO to the NAC P68 + DQA formulations did not significantly alter particle size (Table 1). The mean surface charge of NAC and NAC + DFO associated nanocarriers was moderately positive (+3.67 mV and +6.63 mV, respectively) but each drug-associated formulation had a higher surface charge compared to the blank formulations, which exhibited a slightly negative charge (−0.78 mV) (Table 1).

**Figure 1:**
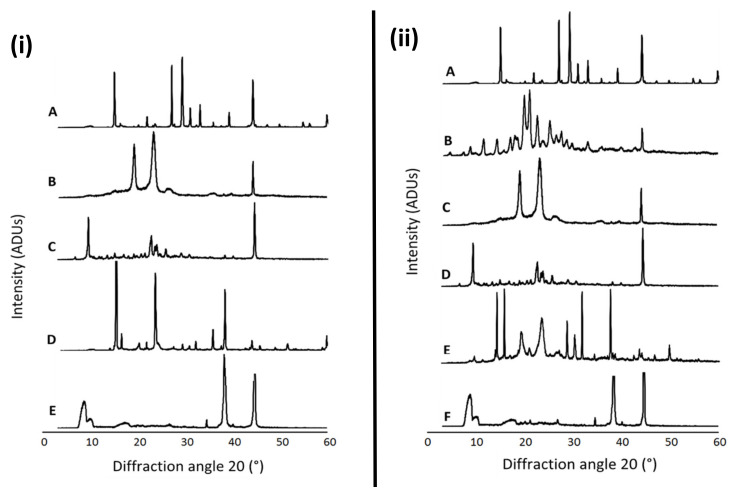
(**i**) P68 + DQA NAC. XRD patterns of (**A**) NAC, (**B**) P68, (**C**) DQA, (**D**) a physical mixture of P68, DQA and NAC in the same ratio as the nanoformulation and (**E**) lyophilized P68 + DQA NAC nanoformulation. (**ii**) P68 + DQA NAC + DFO. XRD patterns of (**A**) NAC, (**B**) DFO, (**C**) P68, (**D**) DQA, (**E**) a physical mixture of P68, DQA, NAC and DFO in the same ratio as the nanoformulation and (**F**) lyophilized P68 + DQA NAC + DFO nanoformulation.

Figure 1 presents the XRD patterns of free NAC, the combination of NAC + DFO, the lyophilized nanoformulations, the physical mixture of the components of each formulation as well as the individual formulation components (P68 and DQA). The spectrum of NAC shows the main peaks at 14.84, 21.8, 26.94, 29.16, 30.94, 32.94, 39.06, 44.12, 59.82°, indicating a high level of crystallinity (Figure 1iA,iiA). DFO also has a crystalline nature, with main peaks at 11.44, 14.16, 17.60, 18.20, 20.76, 20.94, 22.56, 25.20, 27.58, 33.22 and 44.38° (Figure 1iiB). The P68 spectrum exhibits fewer peaks than NAC and DFO, the principal peaks being at 18.94, 23.08, 26.80 and 44.24°, showing some crystalline features (Figure 1iB,iiC). The DQA spectrum also has fewer peaks than NAC and DFO, with the main peaks at 9.42, 22.64, 23.98, 25.72 and 44.5° (Figure 1iC,iiD). The spectra of lyophilized P68 + DQA drug-associated formulations revealed far fewer peaks than its constituent components, with generally only three main peaks for each formulation, at approximately 8.9, 34.7, 38.3 and 44.6°, indicating a more amorphous state (Figure 1iE,iiF). This reduction in peaks was not observed for the physical mixtures of the formulation components (Figure 1iD,iiE).

**Figure 2:**
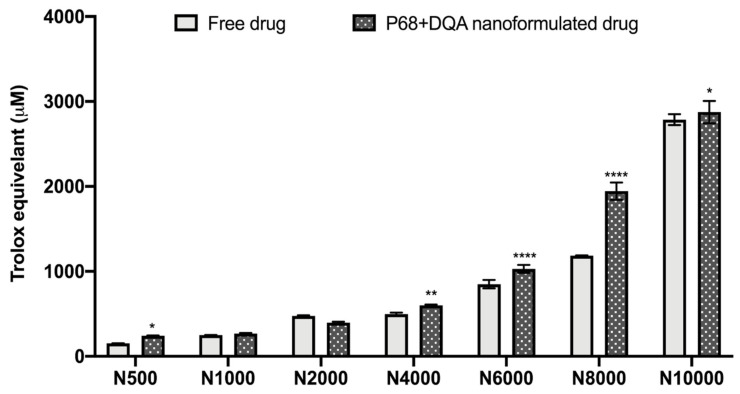
Antioxidant capacity of free and P68 + DQA nanoformulated 500–10,000μM NAC measured by the ferric reducing antioxidant power (FRAP) assay (mean ± S.D., *n* = 6). * represents significance values of nanoformulated drug compared to free drug within the same treatment condition (**** *p* < 0.0001, ** *p* < 0.01, * *p* < 0.05).

The antioxidant capacity of the NAC (500–10,000 μM)-associated P68 + DQA nanocarriers was assessed using the FRAP assay, and compared to the corresponding free drug at each concentration (Figure 2). Significant differences in mean Trolox equivalent antioxidant capacity were observed between the different concentrations of treatments (F(6, 70) = 4088, *p* < 0.0001,) and between the different free or formulated treatment preparations (F(1, 70) = 219.3, *p* < 0.0001) (Figure 2). All concentrations of the P68 + DQA NAC nanocarriers had at least the same antioxidant capacity as the corresponding free-drug concentrations of NAC (Figure 2). The 500 μM (*p* = 0.0222), 4000 μM (*p* = 0.0055), 6000 μM (*p* < 0.0001), 8000 μM (*p* < 0.0001) and 10,000 μM (*p* = 0.0257) concentrations of P68 + DQA NAC exhibited higher antioxidant capacity than free NAC at these concentrations, between 3.19 and 64.15% (Figure 2).

The same concentration range (500–10,000 μM) of free and P68 + DQA NAC was then tested on the SH-SY5Y cell line to evaluate the cytotoxicity of each concentration, using the MTT assay. No cytotoxicity (cell viability below 80%) was observed for any concentration of free or nanoformulated NAC following 24 h treatment (Supplementary Figure S1). However, by 72 h, significant reductions in cell viability were observed for all concentrations apart from 500 and 1000 μM free and P68 + DQA NAC treatments (*p* < 0.0001) (Supplementary Figure S1). The 500 and 1000 μM concentrations of NAC were used for all further assessments and were combined with 50 and 100 μM DFO (respectively) based on the cytotoxicity profile we previously reported for free and P68 + DQA DFO [42].

**Figure 3:**
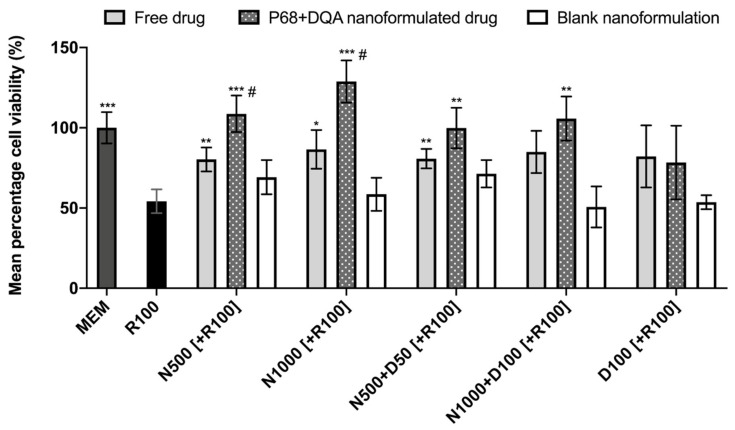
MTT assay results of 3 h pre-treatment with free-drug, P68 + DQA nanoformulated and corresponding blank preparations of either 500 and 1000 μM NAC (N500, N1000), combined NAC with 50 or 100 μM DFO (N500 + D50, N1000 + D100) or 100 μM DFO alone (D100) followed by 24 h treatment with 100 μM rotenone (R100) compared to R100 treatment alone. MEM represents the control condition where cells were only treated with media, no pre-treatment nor R100 treatment (mean ± S.D., *n* = 6). * represents significance values of control or pre-treatment conditions compared to R100 treatment alone (*** *p* < 0.001, ** *p* < 0.01, * *p* < 0.05). # represents significance values of nanoformulated drug compared to free drug within the same treatment condition (# *p* < 0.05).

When comparing the effects of free and P68 + DQA NAC and/or DFO on cell viability following 24 h rotenone treatment, significant differences in mean cell viability were observed between the different treatments (F(16, 51.51) = 18.6, *p* < 0.0001) (Figure 3). The 3 h pre-treatment with P68 + DQA nanocarriers of 500 μM and 1000 μM NAC (*p* = 0.0003 and *p* = 0.0001, respectively), combined 500 μM NAC and 50 μM DFO (*p* = 0.0038) and combined 1000 μM NAC and 100 μM DFO (*p* = 0.0025) significantly protected against the reduction in cell viability induced by 24 h treatment with 100 μM rotenone (Figure 3). Most of the free-drug conditions containing NAC also protected against rotenone induce cytotoxicity (500 μM NAC-*p* = 0.0091, 1000 μM NAC-*p* = 0.028, 500 μM NAC + 50 μM DFO-*p* = 0.0037) but in each case the nanoformulations had a higher mean percentage viability and nanocarriers of 500 and 1000 µM NAC were significantly more protective than the corresponding concentrations of free NAC (35.42% *p* = 0.038 and 48.87% *p* = 0.0122, respectively) (Figure 3). No significant effect was observed with free or P68 + DQA 100 μM DFO pre-treatments. None of the blank formulations were able to protect against rotenone-induced cytotoxicity (Figure 3).

**Figure 4:**
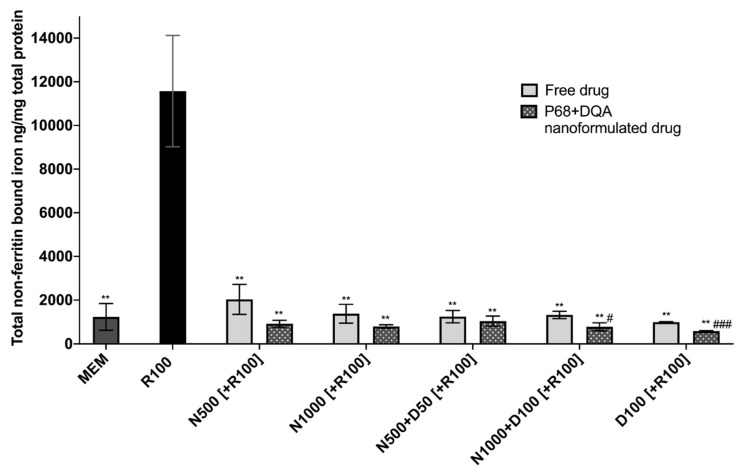
Total non-ferritin-bound iron concentrations resulting from 3 h pre-treatment with free-drug or P68 + DQA nanoformulated preparations of either 500 and 1000 μM NAC (N500, N1000), combined NAC with 50 or 100μM DFO (N500 + D50, N1000 + D100) or 100 μM DFO alone (D100) followed by 24 h treatment with 100 μM rotenone (R100) compared to R100 treatment alone. MEM represents the control condition where cells were only treated with media, no pre-treatment nor R100 treatment (mean ± S.D., *n* = 6). Total non-ferritin-bound iron was calculated using the total iron concentrations obtained from the ferrozine assay minus the mean ferritin concentrations obtained from the ferritin ELISA (see Supplementary Figure S2). * represents the significance of control or pre-treatment conditions compared to R100 treatment alone (** *p* < 0.01). # represents the significance of nanoformulated drug compared to free drug within the same treatment condition (# *p* < 0.05, ## *p* < 0.01).

When evaluating iron status using the ferritin ELISA and ferrozine assay, significant differences in mean non-ferritin-bound iron were observed between the different pre-treatment conditions following 24 h rotenone treatment (F(11, 7.058) = 85.05, *p* < 0.0001) (Figure 4). All P68 + DQA 3 h pre-treatments of NAC and/or DFO were able to protect against 100 μM rotenone-induced increased iron levels (500 μM NAC (*p* = 0.0036), 1000 μM NAC (*p* = 0.0033), 500 μM NAC + 50 μM DFO (*p* = 0.0038), 1000 μM NAC + 100 μM DFO (*p* = 0.0034), 100 μM DFO (*p* = 0.003)) (Figure 4). All concentrations of free NAC and/or DFO also significantly protected against increased iron levels as a result of rotenone treatment (500 μM NAC (*p* = 0.0033), 1000 μM NAC (*p* = 0.0046), 500 μM NAC + 50 μM DFO (*p* = 0.0042), 1000 μM NAC + 100 μM DFO (*p* = 0.0043), 100 μM DFO (*p* = 0.0043)) (Figure 4). A significantly lower level of non-ferritin-bound iron was observed with the P68 + DQA formulations at 1000 μM NAC + 100 μM DFO (40.93%, *p* = 0.0307) and 100 μM DFO (41.60%, *p* = 0.0007) compared to the corresponding free-drug conditions (Figure 4).

**Figure 5:**
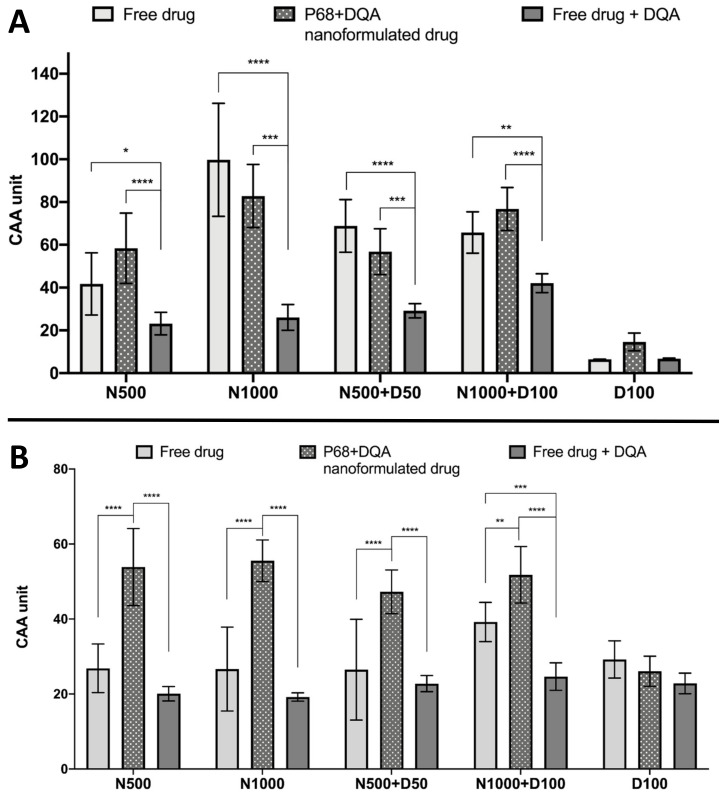
(**A**). Cellular antioxidant activity (CAA) assay results for free-drug, P68 + DQA nanformulated drug and free drug + DQA preparations of 50 or 1000 μM NAC (N500, N1000), combined NAC and 50 or 100 μM DFO (N500 + D50, N1000 + D100) and 100 μM DFO alone (D100) when using 100 μM rotenone as the prooxidant (mean ± S.D., *n* = 6). (**B**). Corresponding CAA results when using 600 μM ABAP as the prooxidant. * represent significance when comparing the P68 + DQA nanoformulated drug to the free-drug and free drug + DQA preparations within the same drug condition (**** *p* < 0.0001, *** *p* < 0.001, ** *p* < 0.01, * *p* < 0.05).

The CAA assay results showed significant differences in cellular antioxidant activity between the different treatment preparation types against 100 μM rotenone (F(2, 75) = 65.07, *p* < 0.0001) and 600 μM ABAP (F(2, 60) = 109.5, *p* < 0.0001) as well as with the different concentrations of NAC and NAC + DFO in the majority of cases (rotenone: F(4, 75) = 60.19, *p* < 0.0001; ABAP: F(4, 75) = 8.121, *p* = 0.0559) (Figure 5). All concentrations of the P68 + DQA preparation of NAC and/or DFO had at least as high cellular antioxidant activity against 100 μM rotenone than the free-drug preparations and, in all cases, the P68 + DQA preparations containing NAC exhibited significantly higher antioxidant activity (83-218%) than the free drug + DQA preparation (500 μM-*p* < 0.0001 and 1000 μM-*p* < 0.0001, 500 μM NAC + 50 μM DFO-*p* = 0.0003 and 1000 μM NAC + 100 μM DFO-*p* < 0.0001) (Figure 5a). The highest antioxidant activity against rotenone was reached at 1000 μM NAC for both the free and P68 + DQA preparations (Figure 5a). All NAC and NAC + DFO free and P68 + DQA treatments resulted in significantly higher cellular antioxidant activity than the corresponding DFO treatments alone (*p* < 0.0001). These results are similar to those seen when using the traditional CAA method, where ABAP was used as the prooxidant, with all P68 + DQA NAC and NAC + DFO treatments also resulting in higher CAA units than P68 + DQA DFO treatment alone (*p* < 0.0001). However, this trend was not observed with the free-drug conditions against ABAP and the P68 + DQA preparations of NAC and NAC + DFO have consistently higher CAA units than free preparations when using ABAP as the prooxidant (Figure 5b). The cellular antioxidant activity of both free and P68 + DQA NAC and NAC + DFO was generally highest when using rotenone as the prooxidant, exhibiting mean CAA units between 42 and 99, compared to 27 and 56 when using ABAP (Figure 5).

**Figure 6:**
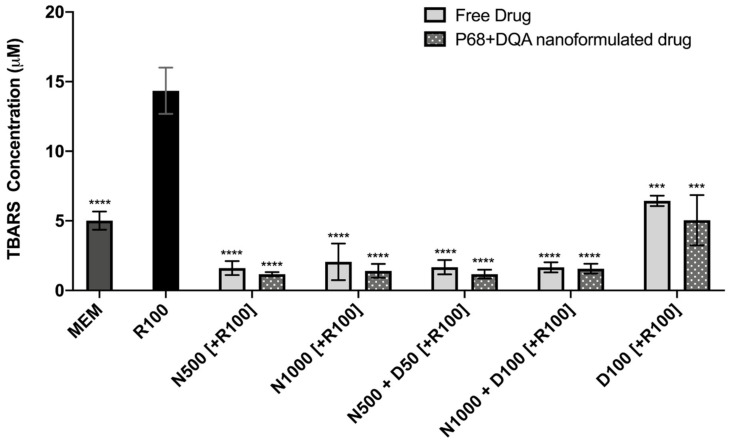
TBARS assay results of 3 h pre-treatment with free-drug or P68 + DQA nanoformulated preparations of either 500 or 10000 μM NAC (N500, N1000), combined NAC and DFO (N500 + D50, N1000 + D100) or 100 μM DFO alone (D100) followed by 24 h treatment with 100 μM rotenone (R100) compared to R100 treatment alone. MEM represents the control condition where cells were only treated with media, no pre-treatment nor R100 treatment (mean ± S.D., *n* = 6). * represents the significance of control or pre- treatment conditions compared to R100 treatment alone (**** *p* < 0.0001, *** *p* < 0.001).

The TBARS assay results showed significant differences in lipid peroxidation between the different pre-treatments (F(11, 22.43) = 128.9, *p* < 0.0001) (Figure 6). Significant protection against rotenone-induced lipid peroxidation was observed with 3 h pre-treatment of both free and P68 + DQA preparations of NAC and/or DFO at all concentrations (*p* ≤ 0.0002). However, the NAC and NAC + DFO pre-treatments generally resulted in greater protection against lipid peroxidation than the 100 μM DFO pre-treatments (*p* < 0.0001 for all free-drug conditions except 1000 μM NAC, where *p* = 0.0023). The NAC and NAC + DFO pre-treatments also resulted in lower lipid peroxidation than at control, in each case resulting in between 59–68% (free-drug preparations) and 69–77% (P68 + DQA nanoformulations) lower TBARS concentrations than at control (*p* < 0.0001 in all cases except 1000 μM free NAC, where *p* = 0.0179) (Figure 6). There was no significant difference in the ability of P68 + DQA nanocarriers and the corresponding free-drug pre-treatments to protect against rotenone-induced lipid peroxidation. However, the mean concentration of TBARS following nanocarrier pre-treatment was generally lower (Figure 6).

**Figure 7:**
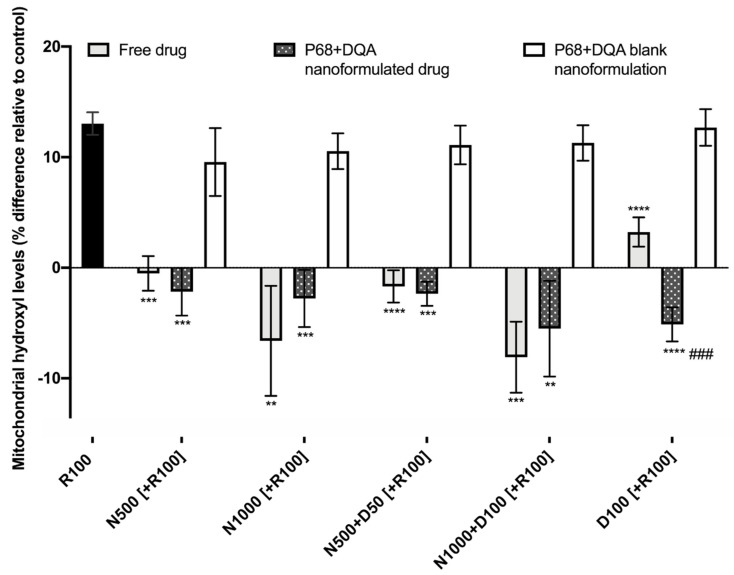
Mitochondrial hydroxyl assay results of 3 h pre-treatment with free, P68 + DQA or corresponding blank formulations of NAC (500 or 1000 μM), combined NAC (500 or 1000 μM) + DFO (50 or 100 μM) or 100 μM DFO alone (D100) followed by 24 h treatment with 100 μM rotenone (R100) (mean ± S.D., *n* = 6). Mitochondrial hydroxyl levels are expressed as the percentage of hydroxyl identified in control cells (SH-SY5Y cells treated with MEM media only, for 24 h). * represents the significance of pre-treatment conditions compared to R100 treatment alone for each marker (**** *p* < 0.0001, *** *p* < 0.001, ** *p* < 0.01). # represents the significance of nanoformulated drug compared to free drug within the same treatment condition (### *p* < 0.001).

When evaluating the ability of free and P68 + DQA formulated NAC and NAC + DFO to protect against increased mitochondrial hydroxyl induced by rotenone, significant differences were observed between the different free and formulated treatments of NAC and/or DFO (F(15, 36.28) = 64.38, *p* < 0.0001) (Figure 7). Figure 7 shows that 24 h treatment with 100 μM rotenone induced a 12% increase in hydroxyl compared with the control (cells treated with MEM only). The 3 h pre-treatment with all concentrations of free and P68 + DQA NAC and/or DFO, but none of the corresponding blank formulations, were able to significantly protect against the rotenone induce rise in hydroxyl, maintaining levels equivalent or within 10% lower than at control (500 μM NAC: free (*p* < 0.0001) and P68 + DQA (*p* = 0.0001), 1000 μM NAC: free (*p* = 0.0065) and P68 + DQA (*p* = 0.0003), 500 μM NAC + 50 μM DFO: free and P68 + DQA (*p* < 0.0001), 1000 μM NAC + 100 μM DFO: free (*p* = 0.0002) and P68 + DQA (*p* = 0.0046), 100 μM DFO: free and P68 + DQA (*p* < 0.0001)) (Figure 7). No significant differences in hydroxyl levels were observed between the free and P68 + DQA NAC and NAC + DFO conditions. However, P68 + DQA 100 μM DFO resulted in significantly lower hydroxyl than free DFO (*p* = 0.0001) (Figure 7).

## 4. Discussion

There is increasing evidence suggesting that NAC is protective in numerous models of PD [22,23,25,26,27,28,58]. Although NAC has shown some promise as a therapeutic candidate in early clinical trials [32,33], the full potential of NAC as a disease-modifying treatment for PD is limited due to issues such as low bioavailability, lack of targeted delivery and insufficient access to the brain [13,34]. Previous studies have shown that the combination of antioxidants with iron chelators may provide a potent strategy against oxidative stress. However, like NAC, currently available iron chelators used to treat iron-overload disorders, including DFO, have similar issues of limited access to the brain and off-target side effects due to lack of targeting which restricts their wider clinical use [59]. Furthermore, it is difficult to achieve the desired effects of combination therapies due to drugs having their own distinct pharmacological profiles [60]. Formulation science using nanocarriers has therefore generated interest for drug and bioactive delivery because nanocarriers can enhance the stability, bioavailability and ability of the associated agents to cross biological membranes as well as co-deliver treatments to targeted locations for synergistic effects [39,40,42,60,61,62]. The aim of this study was to utilise the P68 + DQA micellar nanocarriers previously developed by Mursaleen et al. [42] for the antioxidant NAC, alone or in combination with DFO, and to assess whether these nanoformulations could protect against reduced cell viability, increased free iron and increased oxidative stress induced by a cellular rotenone model of PD.

NAC, alone or combined with DFO, was successfully incorporated into P68 + DQA nanocarriers with high association efficiency (Table 1). This is consistent with previous findings that micelles have high association efficiencies [42,63,64,65]. All P68 + DQA nanocarriers of NAC and NAC + DFO exhibited consistent particle sizes of 130 nm or below (polydispersity indices < 0.24) that should be of sufficient size range to enable passage across the blood–brain barrier (BBB) based on previous reports which suggest that a particle size of < 200 nm is required for brain penetrance [41,61,66]. As it is widely accepted that smaller particle size may be advantageous to assist with brain delivery [41,61,66], our results suggests that these NAC formulations may be more suited as neurological treatments compared to other antioxidants such as curcumin, which have been tested in the same delivery system alone and in combination with DFO [42]. The positive charge of the drug-associated P68 + DQA nanocarriers is consistent with previous reports for formulations using dequalinium [40,42]. Although positive, the mean surface charges of the NAC and NAC + DFO P68 + DQA formulations were relatively neutral (< +7 mV) (Table 1), suggesting that NAC P68 + DQA nanocarriers, with and without the combination of DFO, could access the brain without causing any toxicity to the BBB [41,67,68,69,70,71]. XRD studies revealed that the crystalline nature of NAC and the combination with DFO was suppressed by formulation into P68 + DQA nanocarriers (Figure 1). The concentration ranges selected for NAC (500–10,000 μM) and tested in the FRAP assay were based on and consistent with previous literature [22,72,73,74]. The FRAP results suggested a relationship between increased concentration and increased antioxidant capacity and highlights that at all concentrations the nanocarriers were able to maintain the antioxidant potential and stability of NAC (Figure 2). Together, these results suggest that the P68 + DQA formulations demonstrate suitable characteristics and potential to be utilised for oral or nasal delivery of NAC and enhance absorption of NAC into systemic or neuronal circulation compared to low bioavailability encountered with free NAC [13,34,75].

The cellular testing was carried out using the SH-SY5Y human neuroblastoma cell line as it is the most widely used for in vitro models of PD [49], containing the necessary machinery for dopamine synthesis as shown by its ability to exhibit dopamine-β-hydroxylase activity [76,77,78]. Furthermore, although the SH-SY5Y cell line is of cancerous origin, it retains the majority of the dysregulated genes and cellular mechanistics observed in PD [79]. Ultimately, the 500 and 1000 μM concentrations of NAC were selected for further evaluation as these were the highest concentrations of both the free-drug and P68 + DQA formulations that resulted in no cytotoxicity of SH-SY5Y cells after treatment for up to 72 h (Supplementary Figure S1). The 50 and 100 μM DFO were used in accordance with our previously published data [42].

All NAC and NAC + DFO P68 + DQA nanocarriers were able to significantly protect to at least the same extent as the corresponding free-drug conditions against the 50% reduction in cell viability induced by rotenone (Figure 3), suggesting that all treatments could be protective against cell death exhibited in PD. Both concentrations of P68 + DQA NAC were significantly more protective (35% and 49%, respectively) than the corresponding free NAC (Figure 3), suggesting that the NAC nanoformulations may be superior to free NAC as a potential protective treatment for PD. Unlike the results reported by Mursaleen et al. [42] where the addition of DFO increased the protective effects exhibited by curcumin formulations, the addition of DFO did not result in further protection of NAC (Figure 3). However, the lack of observable effects in both cases could be due to 95–100% cell viability (the same cell viability exhibited by cells treated with MEM media only) being reached in all cases. The P68 + DQA NAC + DFO pre-treatments did, however, result in higher mean cell viabilities than P68 + DQA DFO alone, suggesting that NAC may enhance the effect of DFO since neither free nor formulated DFO alone were able to significantly protect against rotenone-reduced cell viability.

Iron status, total iron and ferritin-bound iron was evaluated, as elevated free iron is thought to be key in the development and progression of PD, mainly due to its ability to drive oxidative stress [1,3,11,12,16,17,74]. Rotenone treatment resulted in an 838% rise of non-ferritin-bound iron compared to control but there was no significant difference in ferritin levels observed between rotenone treatment and control (Figure 4, Supplementary Figure S2). This supports previous reports that rotenone increases levels of free iron [80], as ferritin is the most common source of stored intracellular iron [81] so total non-ferritin-bound iron therefore acts as an indicator of free iron. All free and P68 + DQA NAC and NAC + DFO conditions protected against the rise in total non-ferritin-bound iron induced by rotenone, maintaining control levels (Figure 4). Although not significant in most cases, the P68 + DQA nanoformulations generally resulted in lower levels of total non-ferritin-bound iron compared to the corresponding free-drug conditions (Figure 4). Together, this indicates that the NAC P68 + DQA nanoformulations may protect against the increased levels of free iron evident in PD and therefore protect against iron-induced oxidative stress. The lack of difference in total non-ferritin-bound iron observed between NAC, NAC + DFO and DFO pre-treatments suggests that NAC possesses some inherent iron chelator capacity. This is consistent with previous studies which suggest that NAC can chelate free iron and inhibit the iron-mediated cell death pathway, ferroptosis [74,82,83].

The cellular antioxidant activity results support the idea that NAC, alone and combined with DFO, can protect against PD-related oxidative stress (Figure 5a), as a modified version of the CAA assay developed by Wolfe et al. [53] was carried out, where rotenone was used as the prooxidant in place of ABAP to more closely mimic the oxidative stress present in PD. The cellular antioxidant activity against rotenone of the P68 + DQA preparations of NAC and NAC + DFO, was similar to that of the corresponding free drug in each case (Figure 5a). Free and P68 + DQA NAC and NAC + DFO treatments also exhibited high cellular antioxidant activity against ABAP, although, in most cases, the P68 + DQA preparations were superior (Figure 5b). However, the antioxidant activity was higher for each treatment against rotenone compared to ABAP. The 1000 μM NAC resulted in the most activity for both free and P68 + DQA preparations against rotenone, yielding 154% and 49% higher antioxidant activity than the treatments with the most activity against ABAP (1000 μM NAC + 100 μM DFO for the free-drug preparation and 1000 μM NAC for the P68 + DQA nanoformulation), respectively (Figure 5). This correlates to results shown in vivo that NAC is protective against dopaminergic degeneration induced by rotenone [23]. These data support the notion that NAC may be particularly suited as an antioxidant treatment for PD specifically, since rotenone is the most common neurotoxin model of PD [49] and has been associated with increased incidences of PD in people [7]. These results also support the use of different prooxidants to tailor the CAA assay to the disease model of interest. Unlike the results reported by Mursaleen et al. [42] for curcumin, the addition of DFO to the NAC P68 + DQA nanoformulations did not significantly increase cellular antioxidant activity against rotenone or ABAP (Figure 5). However, the combination with NAC in P68 + DQA nanocarriers enhanced the cellular antioxidant activity of DFO against both rotenone and ABAP by 425% and 99%, respectively. The ability of NAC to generally match or exceed the antioxidant capability of NAC + DFO at both concentrations may be because NAC not only acts as an antioxidant via the glutathione mechanism, converting hydrogen peroxide into water, but it can also chelate iron to some extent directly reducing the formation of hydroxyl via the Fenton reaction [74,82,83].

Similarly, both free and P68 + DQA nanoformulations of NAC and/or DFO were able to protect against the 226% increase in lipid peroxidation induced by rotenone (Figure 6). All the pre-treatments containing NAC resulted in lower lipid peroxidation than observed in the control cells (Figure 6). Although not significantly, the P68 + DQA NAC and NAC + DFO nanoformulations generally resulted in lower lipid peroxidation (between 6 and 32%) than the corresponding free-drug conditions (Figure 6). All NAC and NAC + DFO pre-treatments also resulted in lower lipid peroxidation compared to DFO only pre-treatments, suggesting that combination with NAC may be superior to DFO treatment alone. The mitochondrial hydroxyl assay results further support the ability of these P68 + DQA nanocarriers to protect against oxidative stress as both free and P68 + DQA nanoformulated NAC and NAC + DFO were able to significantly protect against the 12% rise in mitochondrial hydroxyl following rotenone treatment (Figure 7). This indicates that NAC is effective against iron-induced oxidative stress specifically as mitochondrial hydroxyl is the primary oxidant produced by the Fenton reaction in the presence of excess free iron [15,84,85]. Again, no difference was observed between the NAC and NAC + DFO conditions at the same NAC concentrations, supporting the idea that NAC does not benefit from the combination with an iron chelator. Not requiring the addition of an iron chelator to exhibit the most potent protective effects may be of benefit in this case as in practice the combination might lead to more side effects for patients due to the separate pharmacokinetic and safety profiles of NAC and DFO. Although these results also suggest that NAC may be superior to DFO treatment alone, iron chelators have shown some promise in clinical studies of PD [86] and the results of this study may be limited as it is primarily focused on antioxidant outcome measures. Furthermore, previous studies suggest a strong rationale for combination antioxidant and iron chelator therapy as a two-pronged approach to counter the oxidative stress exhibited in PD [42,87], but also because iron has been associated with many of the other hallmarks of PD such as alpha synuclein aggregation [88,89,90], neuroinflammation [91,92] and mitochondrial dysfunction [11,16]. Thus, before making any decisions on the combination of NAC and DFO for PD therapeutics, these formulations will need to be further evaluated to assess these other hallmarks of PD in in vivo disease models.

## 5. Conclusions

Overall, this study demonstrates, for the first time, successful formulation of NAC and NAC + DFO into P68 + DQA nanocarriers for neuronal delivery to protect against oxidative stress in a cellular model of PD. Taken together, the results indicate that NAC and NAC + DFO nanocarriers have the relevant characteristics to access the brain. All NAC and NAC + DFO drug-associated nanocarriers were at least as capable as the corresponding free-drug conditions at protecting against reduced cell viability, increased iron and oxidative stress induced by rotenone. However, in most cases, 1000 μM P68 + DQA NAC exhibited the strongest ability to protect against rotenone, suggesting that NAC alone might be preferential as a potential disease-modifying treatment of PD. As numerous free antioxidants and iron chelators, including NAC and DFO, are limited due to issues such as stability, bioavailability and low brain penetrance, the results presented here further support the notion that the P68 + DQA nanocarrier delivery system may provide a viable solution. Our study demonstrates promising results to support the potential of these formulations for PD therapy. However, further investigations will be required to assess the ability of these formulations to access the brain using a cellular BBB model before they are progressed into the next stage of preclinical testing.

## Figures and Tables

**Table 1:** Hydrodynamic diameter (d), polydispersity index (PDI), surface charge, drug association (DA) and association efficiency (AE) of blank and drug-associated P68 + DQA nanoformulations prepared at 80 °C (mean ± S.D., *n* = 6).

Sample	Contents		*d* (nm)	PDI	Charge (mV)	DA (%)	AE (%)
P68 + DQA (Blank)	P68:	9 mg/mL	25.52 ± 10.25	0.24 ± 0.04	−0.78 ± 0.80	-	-
	DQA:	1 mg/mL					
P68 + DQA NAC	P68:	9 mg/mL	125.67 ± 9.98	0.23 ± 0.05	3.67 ± 0.46	64.88 ± 1.93	92.74 ± 7.54
	DQA: NAC:	1 mg/mL 20 mg/mL					
P68 + DQA NAC + DFO	P68:	9 mg/mL	130.33 ± 11.49	0.24 ± 0.02	6.63 ± 1.44	NAC: 17.53 ± 0.56 DFO: 17.59 ± 0.54	NAC: 98.32 ± 1.44 DFO: 94.36 ± 4.27
	DQA: NAC: DFO:	1 mg/mL 12.4 mg/mL 5 mg/mL					

## Supplementary Materials

The Supplementary Materials are available online at https://www.mdpi.com/2076-3921/9/7/600/s1.

## References

[1] Barodia S.K., Creed R.B., Goldberg M.S. (2017). Parkin and PINK1 functions in oxidative stress and neurodegeneration. Brain Res. Bull..

[2] Tutar Y., Özgür A., Tutar L., Keyshore U. (2013). Role of protein aggregation in neurodegenerative diseases. Neurodegenerative Diseases.

[3] Schapira A.H.V. (2007). Mitochondrial dysfunction in Parkinson’s disease. Cell Death Differ..

[4] Langston W. (1987). MPTP: Insights into the etiology of Parkinson’s disease. Eur. Neurol..

[5] Jenner P., Olanow C.W. (1996). Oxidative stress and the pathogenesis of Parkinson’s disease. Neurology.

[6] Zhou C., Huang Y., Przedborski S. (2008). Oxidative stress in Parkinson’s disease. Ann. N. Y. Acad. Sci..

[7] Tanner C.M., Kamel F., Ross G.W., Hoppin J.A., Goldman S.M., Korell M., Marras C., Bhudhikanok G.S., Kasten M., Chade A.R. (2011). Rotenone, paraquat, and Parkinson’s disease. Environ. Health Perspect..

[8] Camilleri A., Vassallo N. (2014). The centrality of mitochondria in the pathogenesis and treatment of Parkinson’s disease. CNS Neurosci. Ther..

[9] Gautier C.A., Corti O., Brice A. (2014). Mitochondrial dysfunctions in Parkinson’s disease. Rev. Neurol..

[10] Moon H.E., Paek S.H. (2015). Mitochondrial dysfunction in Parkinson’s disease. Exp. Neurobiol..

[11] Jiang H., Wang J., Rogers J., Xie J. (2017). Brain iron metabolism dysfunction in Parkinson’s disease. Mol. Neurobiol..

[12] Kandola K., Bowman A., Birch-Machin M.A. (2015). Oxidative stress–a key emerging impact factor in health, ageing, lifestyle and aesthetics. Int. J. Cosmet. Sci..

[13] Bavarsad Shahripour R., Harrigan M.R., Alexandrov A.V. (2014). N-acetylcysteine (NAC) in neurological disorders: Mechanisms of action and therapeutic opportunities. Brain Behav..

[14] Magesh S., Chen Y., Hu L. (2012). Small Molecule Modulators of K eap1-N rf2-ARE Pathway as Potential Preventive and Therapeutic Agents. Med. Res. Rev..

[15] Thomas C., Mackey M.M., Diaz A.A., Cox D.P. (2009). Hydroxyl radical is produced via the Fenton reaction in submitochondrial particles under oxidative stress: Implications for diseases associated with iron accumulation. Redox Rep..

[16] Simpkins J.W., Dykens J.A. (2008). Mitochondrial mechanisms of estrogen neuroprotection. Brain Res. Rev..

[17] Kroemer G., Reed J.C. (2000). Mitochondrial control of cell death. Nat. Med..

[18] Sian J., Dexter D.T., Lees A.J., Daniel S., Agid Y., Javoy-Agid F., Jenner P., Marsden C.D. (1994). Alterations in glutathione levels in Parkinson’s disease and other neurodegenerative disorders affecting basal ganglia. Ann. Neurol..

[19] Bratic A., Larsson N.G. (2013). The role of mitochondria in aging. J. Clin. Investig..

[20] Wypijewska A., Galazka-Friedman J., Bauminger E.R., Wszolek Z.K., Schweitzer K.J., Dickson D.W., Jaklewicz A., Elbaum D., Friedman A. (2010). Iron and reactive oxygen species activity in parkinsonian substantia nigra. Parkinsonism Relat. Disord..

[21] Barbusiński K. (2009). Fenton reaction-controversy concerning the chemistry. Ecol. Chem. Eng..

[22] Goldstein D.S., Jinsmaa Y., Sullivan P., Sharabi Y. (2017). *N*-Acetylcysteine Prevents the Increase in Spontaneous Oxidation of Dopamine During Monoamine Oxidase Inhibition in PC12 Cells. Neurochem. Res..

[23] Rahimmi A., Khosrobakhsh F., Izadpanah E., Moloudi M.R., Hassanzadeh K. (2015). N-acetylcysteine prevents rotenone-induced Parkinson’s disease in rat: An investigation into the interaction of parkin and Drp1 proteins. Brain Res. Bull..

[24] Perry T.L., Yong V.W., Clavier R.M., Jones K., Wright J.M., Foulks J.G., Wall R.A. (1985). Partial protection from the dopaminergic neurotoxin N-methyl-4-phenyl-1, 2, 3, 6-tetrahydropyridine by four different antioxidants in the mouse. Neurosci. Lett..

[25] Chen C.M., Yin M.C., Hsu C.C., Liu T.C. (2007). Antioxidative and anti-inflammatory effects of four cysteine-containing agents in striatum of MPTP-treated mice. Nutrition.

[26] Park S.W., Kim S.H., Park K.H., Kim S.D., Kim J.Y., Baek S.Y., Chung B.S., Kang C.D. (2004). Preventive effect of antioxidants in MPTP-induced mouse model of Parkinson’s disease. Neurosci. Lett..

[27] Sharma A., Kaur P., Kumar V., Gill K.D. (2007). Attenuation of 1-methyl-4-phenyl-1, 2, 3, 6-tetrahydropyridine induced nigrostriatal toxicity in mice by N-acetyl cysteine. Cell. Mol. Biol..

[28] Clark J., Clore E.L., Zheng K., Adame A., Masliah E., Simon D.K. (2010). Oral N-acetyl-cysteine attenuates loss of dopaminergic terminals in α-synuclein overexpressing mice. PLoS ONE.

[29] Slattery J., Kumar N., Delhey L., Berk M., Dean O., Spielholz C., Frye R. (2015). Clinical trials of N-acetylcysteine in psychiatry and neurology: A systematic review. Neurosci. Biobehav. Rev..

[30] Monti D.A., Zabrecky G., Kremens D., Liang T.W., Wintering N.A., Cai J., Wei X., Bazzan A.J., Zhong L., Bowen B. (2016). *N*-Acetyl Cysteine May Support Dopamine Neurons in Parkinson’s Disease: Preliminary Clinical and Cell Line Data. PLoS ONE.

[31] Reyes R.C., Cittolin-Santos G.F., Kim J.E., Won S.J., Brennan-Minnella A.M., Katz M., Glass G.A., Swanson R.A. (2016). Neuronal glutathione content and antioxidant capacity can be normalized in situ by N-acetyl cysteine concentrations attained in human cerebrospinal fluid. Neurotherapeutics.

[32] Monti D.A., Zabrecky G., Kremens D., Liang T.W., Wintering N.A., Bazzan A.J., Zhong L., Bowens B.K., Chervoneva I., Intenzo C. (2019). *N*-Acetyl Cysteine Is Associated With Dopaminergic Improvement in Parkinson’s Disease. Clin. Pharmacol. Ther..

[33] Katz M., Won S.J., Park Y., Orr A., Jones D.P., Swanson R.A., Glass G.A. (2015). Cerebrospinal fluid concentrations of N-acetylcysteine after oral administration in Parkinson’s disease. Parkinsonism Relat. Disord..

[34] Cotgreave I.A., Moldéus P. (1987). Methodologies for the analysis of reduced and oxidized N-acetylcysteine in biological systems. Biopharm. Drug Dispos..

[35] Tse H.N., Raiteri L., Wong K.Y., Yee K.S., Ng L.Y., Wai K.Y., Loo C.K., Chan M.H. (2013). High-dose N-acetylcysteine in stable COPD: The 1-year, double-blind, randomized, placebo-controlled HIACE study. Chest.

[36] Ferreira L.F., Campbell K.S., Reid M.B. (2011). N-acetylcysteine in handgrip exercise: Plasma thiols and adverse reactions. Int. J. Sport Nutr. Exerc. Metab..

[37] Arstall M.A., Yang J., Stafford I., Betts W.H., Horowitz J.D. (1995). N-acetylcysteine in combination with nitroglycerin and streptokinase for the treatment of evolving acute myocardial infarction: Safety and biochemical effects. Circulation.

[38] Garrido M., Tereshchenko Y., Zhevtsova Z., Taschenberger G., Bähr M., Kügler S. (2011). Glutathione depletion and overproduction both initiate degeneration of nigral dopaminergic neurons. Acta Neuropathol..

[39] Masserini M. (2013). Nanoparticles for brain drug delivery. ISRN Biochem..

[40] Zupančič S., Kocbek P., Zariwala M.G., Renshaw D., Gul M.O., Elsaid Z., Taylor K.M., Somavarapu S. (2014). Design and development of novel mitochondrial targeted nanocarriers, DQAsomes for curcumin inhalation. Mol. Pharm..

[41] Zhou Y., Peng Z., Seven E.S., Leblanc R.M. (2018). Crossing the blood-brain barrier with nanoparticles. J. Control. Release.

[42] Mursaleen L., Somavarapu S., Zariwala M.G. (2020). Deferoxamine and Curcumin Loaded Nanocarriers Protect Against Rotenone-Induced Neurotoxicity. J. Parkinson’s Dis..

[43] Srivastava R.K., Rahman Q., Kashyap M.P., Lohani M., Pant A.B. (2011). Ameliorative effects of dimetylthiourea and N-acetylcysteine on nanoparticles induced cyto-genotoxicity in human lung cancer cells-A549. PLoS ONE.

[44] Dou Q.L., Wei Y.Y., Gu Y.N., Zheng H. (2017). Investigating the therapeutic effects of N-acetylcysteine decorated poly (L-lactic acid) nanoparticles on transfusion induced acute lung injury. J. Biomater. Tissue Eng..

[45] Hamedinasab H., Rezayan A.H., Mellat M., Mashreghi M., Jaafari M.R. (2020). Development of chitosan-coated liposome for pulmonary delivery of N-acetylcysteine. Int. J. Biol. Macromol..

[46] Weissig V., Lasch J., Erdos G., Meyer H.W., Rowe T.C., Hughes J. (1998). DQAsomes: A novel potential drug and gene delivery system made from Dequalinium. Pharm. Res..

[47] Anon A.O.A.C. (2012). Standard method performance requirements for in vitro determination of total antioxidant activity in foods, beverages, food ingredients, and dietary supplements. J. AOAC Int..

[48] Yusof H.I., Owusu-Apenten R., Nigam P.S. (2018). Determination of iron (III) reducing antioxidant capacity for manuka honey and comparison with ABTS and other methods. J. Adv. Biol. Biotech..

[49] Xicoy H., Wieringa B., Martens G.J. (2017). The SH-SY5Y cell line in Parkinson’s disease research: A systematic review. Mol. Neurodegener..

[50] Kim S., Park S.E., Sapkota K., Kim M.K., Kim S.J. (2011). Leaf extract of *Rhus verniciflua* Stokes protects dopaminergic neuronal cells in a rotenone model of Parkinson’s disease. J. Pharm. Pharmacol..

[51] Martins J.B., Bastos M.D.L., Carvalho F., Capela J.P. (2013). Differential effects of methyl-4-phenylpyridinium ion, rotenone, and paraquat on differentiated SH-SY5Y cells. J. Toxicol..

[52] Zariwala M.G., Somavarapu S., Farnaud S., Renshaw D. (2013). Comparison study of oral iron preparations using a human intestinal model. Sci. Pharm..

[53] Wolfe K.L., Liu R.H. (2007). Cellular antioxidant activity (CAA) assay for assessing antioxidants, foods, and dietary supplements. J. Agric. Food Chem..

[54] Chen L., Hambright W.S., Na R., Ran Q. (2015). Ablation of the ferroptosis inhibitor glutathione peroxidase 4 in neurons results in rapid motor neuron degeneration and paralysis. J. Biol. Chem..

[55] Hu B., Ting Y., Zeng X., Huang Q. (2013). Bioactive peptides/chitosan nanoparticles enhance cellular antioxidant activity of (−)-epigallocatechin-3-gallate. J. Agric. Food Chem..

[56] Dutta R.K., Nenavathu B.P., Gangishetty M.K., Reddy A.V.R. (2012). Studies on antibacterial activity of ZnO nanoparticles by ROS induced lipid peroxidation. Colloids Surf. B Biointerfaces.

[57] Chakraborti S., Chakraborty S., Saha S., Manna A., Banerjee S., Adhikary A., Sarwar S., Hazra T.K., Das T., Chakrabarti P. (2017). PEG-functionalized zinc oxide nanoparticles induce apoptosis in breast cancer cells through reactive oxygen species-dependent impairment of DNA damage repair enzyme NEIL2. Free Radic. Biol. Med..

[58] Soto-Otero R., Méndez-Álvarez E., Hermida-Ameijeiras Á., Muñoz-Patiño A.M., Labandeira-Garcia J.L. (2000). Autoxidation and neurotoxicity of 6-hydroxydopamine in the presence of some antioxidants: Potential implication in relation to the pathogenesis of Parkinson’s disease. J. Neurochem..

[59] Liu Z., Purro M., Qiao J., Xiong M.P. (2017). Multifunctional polymeric micelles for combining chelation and detection of iron in living cells. Adv. Healthc. Mater..

[60] Anselmo A.C., Mitragotri S. (2016). Nanoparticles in the clinic. Bioeng. Transl. Med..

[61] Grabrucker A.M., Ruozi B., Belletti D., Pederzoli F., Forni F., Vandelli M.A., Tosi G. (2016). Nanoparticle transport across the blood brain barrier. Tissue Barriers.

[62] Saraiva C., Praça C., Ferreira R., Santos T., Ferreira L., Bernardino L. (2016). Nanoparticle-mediated brain drug delivery: Overcoming blood–brain barrier to treat neurodegenerative diseases. J. Control. Release.

[63] Zhang P., Hu L., Yin Q., Zhang Z., Feng L., Li Y. (2012). Transferrin-conjugated polyphosphoester hybrid micelle loading paclitaxel for brain-targeting delivery: Synthesis, preparation and in vivo evaluation. J. Control. Release.

[64] Elezaby R.S., Gad H.A., Metwally A.A., Geneidi A.S., Awad G.A. (2017). Self-assembled amphiphilic core-shell nanocarriers in line with the modern strategies for brain delivery. J. Control. Release.

[65] Rakotoarisoa M., Angelova A. (2018). Amphiphilic nanocarrier systems for curcumin delivery in neurodegenerative disorders. Medicines.

[66] Cruz L.J., Stammes M.A., Que I., van Beek E.R., Knol-Blankevoort V.T., Snoeks T.J., Löwik C.W. (2016). Effect of PLGA NP size on efficiency to target traumatic brain injury. J. Control. Release.

[67] Lockman P.R., Koziara J.M., Mumper R.J., Allen D.D. (2004). Nanoparticle surface charges alter blood–brain barrier integrity and permeability. J. Drug Target..

[68] Choi C.H.J., Alabi C.A., Webster P., Davis M.E. (2010). Mechanism of active targeting in solid tumors with transferrin-containing gold nanoparticles. Proc. Natl. Acad. Sci. USA.

[69] Wiley D.T., Webster P., Gale A., Davis M.E. (2013). Transcytosis and brain uptake of transferrin-containing nanoparticles by tuning avidity to transferrin receptor. Proc. Natl. Acad. Sci. USA.

[70] Huang X., Li L., Liu T., Hao N., Liu H., Chen D., Tang F. (2011). The shape effect of mesoporous silica nanoparticles on biodistribution, clearance, and biocompatibility in vivo. ACS Nano.

[71] Bramini M., Ye D., Hallerbach A., Nic Raghnaill M., Salvati A., Aberg C., Dawson K.A. (2014). Imaging approach to mechanistic study of nanoparticle interactions with the blood–brain barrier. ACS Nano.

[72] Martínez M.A., Rodríguez J.L., Lopez-Torres B., Martínez M., Martínez-Larrañaga M.R., Maximiliano J.E., Anadón A., Ares I. (2020). Use of human neuroblastoma SH-SY5Y cells to evaluate glyphosate-induced effects on oxidative stress, neuronal development and cell death signaling pathways. Environ. Int..

[73] Ganguly U., Ganguly A., Sen O., Ganguly G., Cappai R., Sahoo A., Chakrabarti S. (2019). Dopamine cytotoxicity on SH-SY5Y cells: Involvement of α-synuclein and relevance in the neurodegeneration of sporadic Parkinson’s disease. Neurotox. Res..

[74] Do Van B., Gouel F., Jonneaux A., Timmerman K., Gele P., Petrault M., Bastide M., Laloux C., Moreau C., Bordet R. (2016). Ferroptosis, a newly characterized form of cell death in Parkinson’s disease that is regulated by PKC. Neurobiol. Dis..

[75] Savjani K.T., Gajjar A.K., Savjani J.K. (2012). Drug solubility: Importance and enhancement techniques. ISRN Pharm..

[76] Biedler J.L., Roffler-Tarlov S., Schachner M., Freedman L.S. (1978). Multiple Neurotransmitter Synthesis by Human Neuroblastoma Cell Lines and Clones. Cancer Res..

[77] Påhlman S., Ruusala A.-I., Abrahamsson L., Mattsson M.E.K., Esscher T. (1984). Retinoic acid-induced differentiation of cultured human neuroblastoma cells: A comparison with phorbolester-induced differentiation. Cell Differ..

[78] Ross R.A., Biedler J.L. (1985). Presence and Regulation of Tyrosinase Activity in Human Neuroblastoma Cell Variants in Vitro. Cancer Res..

[79] Krishna A., Biryukov M., Trefois C., Antony P.M.A., Hussong R., Lin J., Heinäniemi M., Glusman G., Köglsberger S., Boyd O. (2014). Systems genomics evaluation of the SH-SY5Y neuroblastoma cell line as a model for Parkinson’s disease. BMC Genomics.

[80] Mouhape C., Costa G., Ferreira M., Abin-Carriquiry J.A., Dajas F., Prunell G. (2019). Nicotine-induced neuroprotection in rotenone in vivo and in vitro models of Parkinson’s disease: Evidences for the involvement of the labile iron pool level as the underlying mechanism. Neurotox. Res..

[81] Ropele S., Enzinger C., Fazekas F. (2017). Iron mapping in multiple sclerosis. Neuroimaging Clin..

[82] Wongjaikam S., Kumfu S., Khamseekaew J., Chattipakorn S.C., Chattipakorn N. (2017). Restoring the impaired cardiac calcium homeostasis and cardiac function in iron overload rats by the combined deferiprone and N-acetyl cysteine. Sci. Rep..

[83] Hjortsrø E., Fomsgaard J.S., Fogh-Andersen N. (1990). Does N-acetylcysteine increase the excretion of trace metals (calcium, magnesium, iron, zinc and copper) when given orally?. Eur. J. Clin. Pharmacol..

[84] Costa-Mallen P., Gatenby C., Friend S., Maravilla K.R., Hu S.C., Cain K.C., Agarwal P., Anzai Y. (2017). Brain iron concentrations in regions of interest and relation with serum iron levels in Parkinson disease. J. Neurol. Sci..

[85] Zucca F.A., Segura-Aguilar J., Ferrari E., Muñoz P., Paris I., Sulzer D., Sarna T., Casella L., Zecca L. (2017). Interactions of iron, dopamine and neuromelanin pathways in brain aging and Parkinson’s disease. Prog. Neurobiol..

[86] Devos D., Moreau C., Devedjian J.C., Kluza J., Petrault M., Laloux C., Duhamel A. (2014). Targeting chelatable iron as a therapeutic modality in Parkinson’s disease. Antioxid. Redox Signal..

[87] Sripetchwandee J., Pipatpiboon N., Chattipakorn N., Chattipakorn S. (2014). Combined therapy of iron chelator and antioxidant completely restores brain dysfunction induced by iron toxicity. PLoS ONE.

[88] Brundin P., Dave K.D., Kordower J.H. (2017). Therapeutic approaches to target alpha-synuclein pathology. Exp. Neurol..

[89] Hare D.J., Double K.L. (2016). Iron and dopamine: A toxic couple. Brain.

[90] Ostrerova-Golts N., Petrucelli L., Hardy J., Lee J.M., Farer M., Wolozin B. (2000). The A53T α-synuclein mutation increases iron-dependent aggregation and toxicity. J. Neurosci..

[91] Urrutia P., Aguirre P., Esparza A., Tapia V., Mena N.P., Arredondo M., González-Billault C., Núñez M.T. (2013). Inflammation alters the expression of DMT1, FPN1 and hepcidin, and it causes iron accumulation in central nervous system cells. J. Neurochem..

[92] Saleppico S., Mazzolla R., Boelaert J.R., Puliti M., Barluzzi R., Bistoni F., Blasi E. (1996). Iron regulates microglial cell-mediated secretory and effector functions. Cell. Immunol..

